# Analysis of cyclic deformation of the aortic annulus by cine-MRI

**DOI:** 10.1186/1532-429X-15-S1-P277

**Published:** 2013-01-30

**Authors:** Yang Chul Boering, Sebastian Gruenig, Florian Bönner, Jan Balzer, Mirja Neizel, Marc W Merx, Tobias Zeus, Burkhard Sievers, Malte Kelm

**Affiliations:** 1 Cardiology, Heinrich Heine University, Duesseldorf, Germany

## Background

Imaging of the aortic annulus is paramount for the success of transcatheter aortic valve implantation (TAVI) to obviate prosthesis size mismatch. The aortic annulus is a dynamic structure with alterations of its ellipsoid shape during the cardiac cycle. The objective of this study was to investigate with cine-MRI the changes of the sagittal and coronal diameter of the aortic annulus and whether the point in time of measurement is relevant for prosthesis size selection.

## Methods

40 patients (17 males, 84.3 ± 6.7 years) with severe aortic stenosis were studied with a 1.5 Tesla MR Scanner (Achieva, Philips, The Netherlands) before TAVI. Cine-MRI were acquired in coronal axis and sagittal (3-chamber) view of the left ventricle and the aortic annulus diameter was measured at maximum systole and minimal diastole in each view and the mean annulus diameter was calculated for each phase. Additionally an eccentricity index (EI), the ratio of maximum and minimum diameter was calculated as a measure of circularity of the annulus.

## Results

The maximum diameter of the aortic annulus was measured at systole for both sagittal (mean 23.7±1.7 mm) and coronal axis (mean 25.0±1.9 mm), as the minimal diameter was measured at diastole for the sagittal (mean 21.1±1.9 mm) and the coronal view (mean 23.6±2.4 mm). The degree of deformation was apparently greater in the sagittal direction than in the coronal extent (2.6±0.6 mm vs. 1.5±0.8 mm, respectively, p<0.01). This resulted in a more circular shape of the annulus during systole, reflected by a decreased EI in comparison to diastole (EI=1.06±04 vs. EI=1.12±0.08, respectively, p<0.01). Also the mean annulus diameter was calculated 2 mm smaller than in systole (22.33 ±2.05 mm vs. 24.34±1.75 mm).

## Conclusions

The deformation of the annulus during the cardiac cycle, specifically in the sagittal dimension results in diameter variations of at least 2 mm. It is reasonable that the assessment of the annulus size should be performed in systole, when the annulus is also more circular than in diastole.

## Funding

No disclosures.

